# Supplementary material for: Detection of Triacetone Triperoxide (TATP) Precursors with an Array of Sensors Based on MoS2/RGO Composites
Source: Sensors (Basel). 2019 Mar 13;19(6):1281. doi: 10.3390/s19061281 (PMC6472037; doi:10.3390/s19061281)
Supplement: Supplementary file 1 [file sensors-19-01281-s001.pdf]

sensors

Supporting Information

Detection of Triacetone Triperoxide (TATP) Precursors With an Array of Sensors based on MoS_2/RGO Composites

Qihua Sun [1], Zhaofeng Wu [1,2,*], Haiming Duan [1,*] and Dianzeng Jia[2,*]

1 School of Physics Science and Technology, Xinjiang University, Urumqi, Xinjiang 830046, China; SUNQIHUA520@163.com

2 Key Laboratory of Energy Materials Chemistry, Ministry of Education, Key Laboratory of Advanced Functional Materials, Xinjiang University, Urumqi, Xinjiang 830046, China

* Correspondings: wzf911@mail.ustc.edu.cn (Z.W), dhm@xju.edu.cn (H.D), jdz@xju.edu.cn (D.J); Fax: +86-991-858-2401 (H.D.)

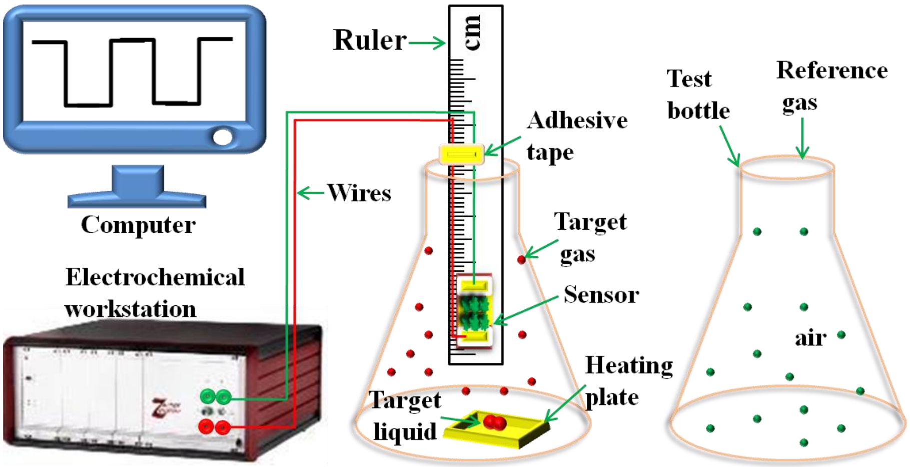

Figure S1. Schematic diagram of gas sensitivity test.

First, the liquid target analyte droplets are added to the heating plate to produce the target analyte vapor with the required concentration by heating (Figure S1). Second, sensor chips and wires are fixed on rulers by tapes and fixtures in order to prevent the shaking of wires and obtain stable response data. Therefore, the sensor can be stably moved into different atmosphere (target gas and reference gas) by moving the ruler from the target gas to the reference gas. In this way, stable gas sensitive data can be recorded by an electrochemical workstation at room temperature.

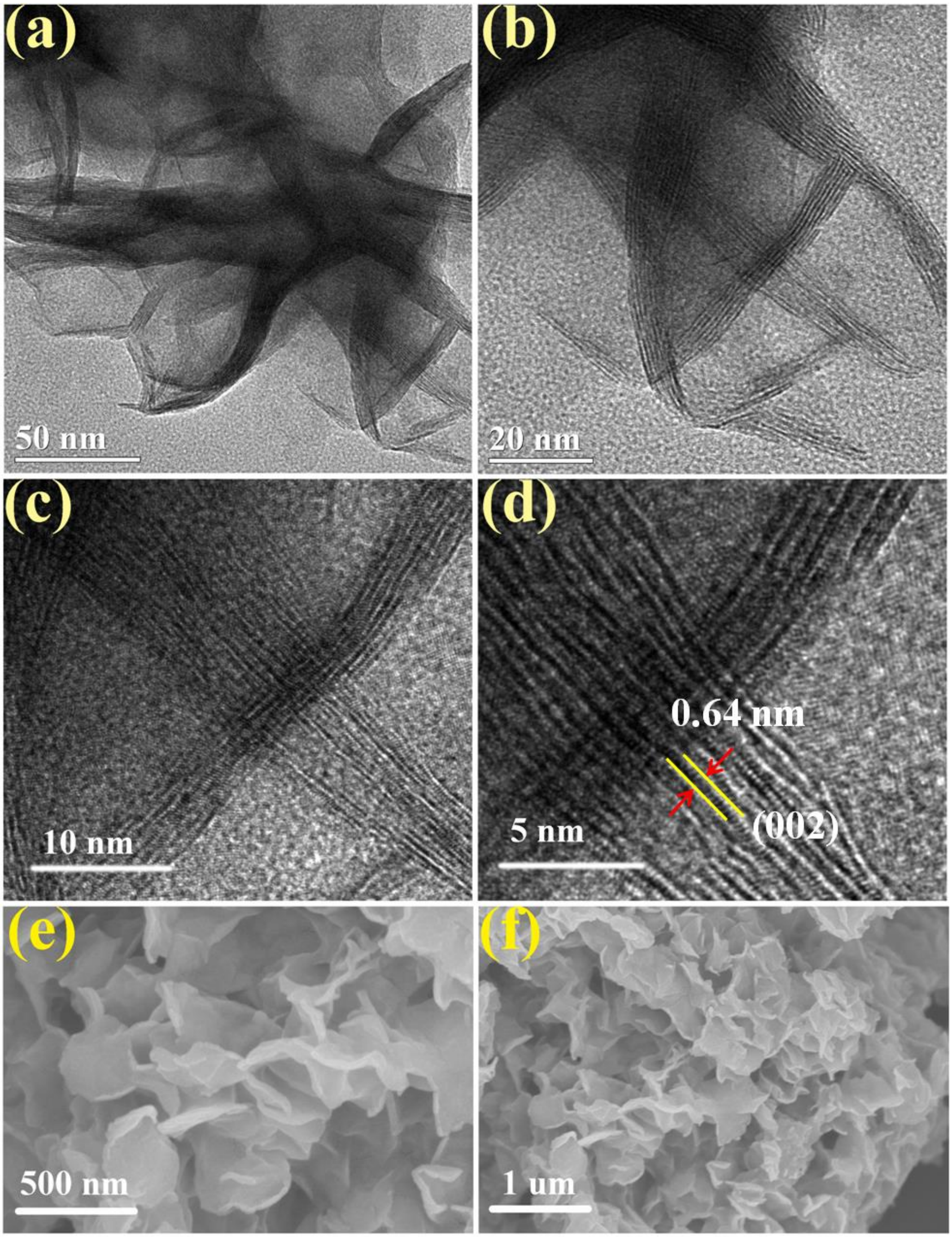

Figure S2. (**a**–**d**) TEM images with different magnification and (**e**,**f**) SEM images of pure MoS_2.

As shown by the yellow lines in the Figure S2d, the crystalline interplanar spacing of pure MoS_2, and the average interplanar spacing from the ten spacing was calculated to be 0.64 nm. The 0.64 nm of interplanar spacing was attributed to the (002) planes of MoS_2.

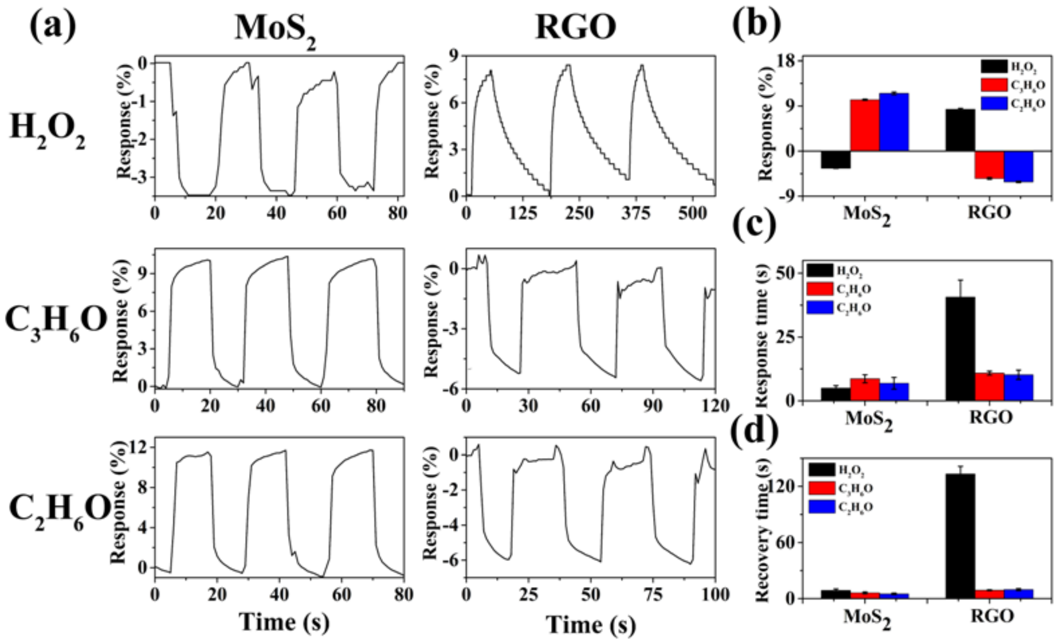

Figure S3. (**a**) Dynamic response curves of the sensors based on pure MoS_2 and pure RGO to 1000 ppm of H_2O_2, C_3H_6O, C_2H_6O vapors at room temperature, statistical graph of (**b**) average response, (**c**) response time and (**d**) recovery time.

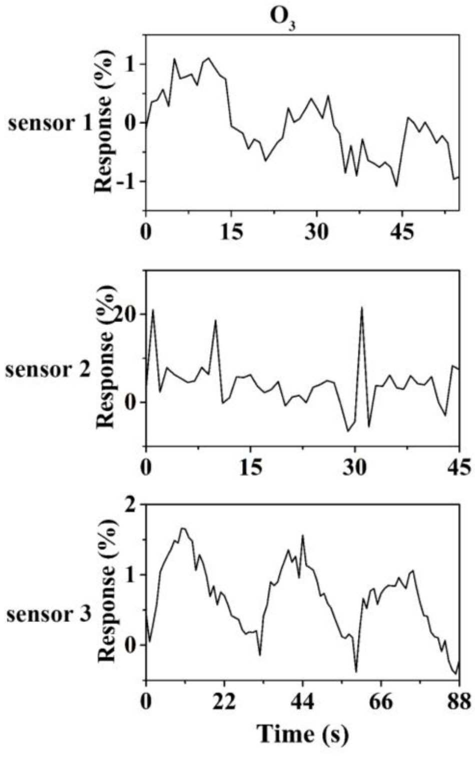

Figure S4. Dynamic sensing curves of the sensor 1, 2 and 3 to 50 ppm of O_3 at room temperature.

A possible cause of a large drift in the O_3 baseline is that O_3 is a strong oxidizing gas, after the sensor adsorbs O_3, the desorption process is slow, and the desorption process is not complete, which will cause the baseline to drift. For solutions that encounter baseline drift, the desorption process can be thoroughly achieved by increasing the time of desorption, but this will increase the recovery time of the sensor. Or it is possible to accelerate the desorption process of gas by ultraviolet irradiation. This method will increase the cost of production.
